# Socioeconomic inequalities and smoking cessation among Hungarian adults: evidence from the European health interview survey

**DOI:** 10.3389/fpubh.2026.1739272

**Published:** 2026-02-03

**Authors:** Battamir Ulambayar, Amr Sayed Ghanem, Ágnes Tóth, Attila Csaba Nagy

**Affiliations:** 1 Department of Epidemiology, Faculty of Health Sciences, University of Debrecen, Debrecen, Hungary; 2 Department of Nursing and Integrative Health Sciences, Faculty of Health Sciences, University of Debrecen, Debrecen, Hungary

**Keywords:** demographic factors, European health interview survey, Hungary, smoking cessation, socio-economic factors

## Abstract

**Background:**

Smoking remains a major public health challenge in Hungary, where prevalence exceeds European and global averages despite comprehensive tobacco control measures. This study examines the socio-demographic and economic factors associated with smoking cessation among Hungarian adults.

**Methods:**

A cross-sectional analysis was conducted using data from the 2019 European Health Interview Survey (EHIS). The study included 2,598 participants with a history of smoking, categorized as current smokers (58.4%) or former smokers (41.6%). Chi-square tests and weighted logistic regression models, including interaction terms, were applied to assess associations between smoking cessation and demographic and socioeconomic characteristics such as gender, age, education, income, employment, urbanization, and marital status.

**Results:**

Females, younger adults, those in remote areas, and unmarried, divorced, or widowed individuals had lower odds of smoking cessation, while higher education and higher-middle income were associated with higher odds of smoking cessation. Interaction analyses indicated that females, individuals with higher education, and those with greater income levels were associated with higher odds of smoking cessation, particularly among younger and middle-aged adults.

**Conclusion:**

These findings highlight the persistence of socioeconomic inequalities in smoking cessation within the Hungarian population and underscore the need for targeted interventions that address gender, educational, and regional disparities.

## 1 Introduction

Smoking remains one of the leading causes of morbidity and mortality worldwide, contributing to cardiovascular disease (1), cancer (2), respiratory disease (3), in addition to a wide range of chronic diseases (4). Since more than 8 million people die from smoking each year globally, over 7 million due to direct tobacco use and 1.3 million from second-hand smoke exposure among non-smokers, smoking is referred to as a global epidemic (5). Despite global efforts to reduce tobacco use through public health campaigns, policy interventions, and cessation programs, smoking remains a major public health challenge worldwide (6, 7).

Hungary, a Central European country facing persistent social and economic inequalities, has long struggled with high smoking rates compared to many European countries (8). A 2007 national survey found that 36.1% of Hungarian adults smoked cigarettes, with higher rates among males (40.6%) compared to females (31.7%), and the risk factors for smoking included male gender, younger age, lower education, lower socioeconomic status, and parental smoking (9). A more recent study analyzing data from 2010 to 2018 reported decreased rates in overall smoking prevalence, with 24.4% of males and 24.2% of females smoking every day. However, this study also noted a concerning trend of increasing smoking rates from 2016 onwards, particularly among women, and the prevalence of smoking in Hungary significantly exceeds European and global averages across all age groups (10). The country has implemented significant anti-smoking measures in recent years. In 2011, a comprehensive smoking ban was introduced in public places, workplaces, and public transport, leading to an estimated 1,700 postponed deaths and 16,000 life years saved annually (11). The country established the Quitline, a telephone-based smoking cessation service in 2012, which conducted over 12,000 calls between 2015 and 2020, offering tailored support for smoking cessation (12). A major regulatory change in 2013 restricted tobacco sales to designated National Tobacco Shops, reducing retail density by 85%. This policy led to a measurable decline in adolescent smoking prevalence (13). Hungary also implements a smoking cessation support program to reduce smoking, but it remains the country's biggest public health problem.

Smoking cessation, the process of quitting tobacco use, is affected by a multifaceted interplay of individual, social, and environmental factors (14). At the individual level, smoking cessation is shaped by multiple interrelated factors, including psychological aspects such as self-efficacy and motivation, as well as biological factors like nicotine dependence (15). Psychosocial factors, including family support, peer influence, and cultural attitudes toward smoking, further shape an individual's ability to quit (16).

We hypothesize that, in the Hungarian context, smoking cessation is significantly associated with socioeconomic inequalities. We aim to identify the key demographic and socio-economic factors associated with smoking cessation in the Hungarian population, using nationally representative data from the European Health Interview Survey 2019 (EHIS 2019). These insights are essential for informing evidence-based strategies to reduce tobacco-related harm and support Hungary's efforts to meet global health targets under the WHO Framework Convention on Tobacco Control (FCTC), which calls for a 30% reduction in tobacco use by 2025, implementation of comprehensive smoke-free laws, and expanded access to cessation support services. Understanding these relationships can inform more equitable and effective tobacco control policies tailored to Hungary's population.

## 2 Methods

### 2.1 Study design and data

This study employed a cross-sectional design to investigate the socio-economic and demographic factors associated with smoking cessation in Hungary, utilizing data from the 2019 European Health Interview Survey (EHIS). The EHIS, conducted under Eurostat's supervision, is a standardized, population-based survey designed to collect comparable health-related data across European countries. In Hungary, the survey was administered by the Hungarian Central Statistical Office, targeting a nationally representative sample of 5,603 individuals aged 15 years and older, selected via stratified random sampling to reflect the country's demographic and geographic diversity (17). Data were collected through face-to-face interviews and phone calls in 2019, using a structured questionnaire. For this analysis, we focused on participants with a smoking history, excluding those who reported never smoking (*n* = 3,005, 54.4%) to isolate factors associated with cessation among ever-smokers. The final study population comprised 2,531 participants, categorized into two groups: active smokers (*n* = 1,478, 58.4%), defined as current smokers regardless of quit attempts, and successful cessation (*n* = 1,053, 41.6%), defined as former smokers who had ceased smoking at the time of the survey.

### 2.2 Variables

Active smokers included individuals who had never attempted to quit as well as those who had made unsuccessful attempts within the past year. Smoking cessation was defined based on self-reported cigarette smoking status at the time of the EHIS 2019 interview. Former smokers were individuals who reported that they did not smoke cigarettes at the time of the survey and who had quit smoking either more than 1 year prior to the interview or within the past year but for a duration exceeding 1 month. This definition reflects point prevalence abstinence at the time of data collection. Abstinence was assessed for cigarette smoking only; the EHIS dataset does not allow for consistent classification of abstinence from other nicotine-containing products, such as e-cigarettes or heated tobacco products.

Independent variables were selected based on established associations with smoking cessation in prior literature and their relevance to Hungary's socio-economic landscape. Demographic variables included gender, age (categorized as 15–17, 18–34, 35–64, and ≥65 years) to reflect distinct life stages and smoking trends), and age at smoking initiation (before 16 years or 16 years and older, as earlier onset may signal stronger nicotine dependence). Socio-economic variables encompassed education level (primary, secondary, or high), income level (divided into quintiles as low, lower-middle, middle, higher-middle, and high, based on self-reported household income adjusted for household size), employment status (classified as regular employment, irregular employment, retired/disabled, or student/childcare, capturing economic stability and time availability), degree of urbanization (urban, suburban, rural, or remote, derived from population density and geographic classifications to reflect access to cessation resources), and marital status (registered marriage, unmarried partner, no marriage, divorced, or widowed, serving as a proxy for social support). Additional covariates included health status (self-reported as good/excellent, satisfactory, or bad/very bad to assess health-related motivation for quitting) and long-term illness (yes or no, based on diagnoses of chronic conditions that might influence cessation intent).

### 2.3 Statistical analysis

Chi-squared tests were used to assess bivariate associations between smoking cessation status and each independent variable (*p* < 0.05). Variables showing significant associations were subsequently entered into weighted multivariable logistic regression models to estimate adjusted odds of successful smoking cessation, expressed as odds ratios (OR) with 95% confidence intervals (CI). Smoking cessation was coded as a binary outcome (1 = successful cessation, 0 = active smoker). All analyses were conducted using survey weights provided by the EHIS 2019 to account for the complex sampling design, including unequal probabilities of selection, non-response, and post-stratification. Weighted data were applied consistently across all analyses, including descriptive statistics, chi-square tests, and regression models. Survey-specific analytical procedures were used to appropriately account for the complex survey design. Model performance was evaluated using the Area Under the Receiver Operating Characteristic (ROC) Curve. Interaction terms between key socio-demographic factors were tested to assess subgroup differences. All analyses were performed using Stata IC 18.0 (18).

## 3 Results

Table 1 summarizes the associations between smoking cessation and socio-economic factors. Cessation was significantly associated with age, education, income, employment, urbanization, and marital status (all *p* < 0.001). Higher cessation rates were observed in older age groups, from minimal success among young adults to the highest rates among those aged ≥65 years. Individuals who started smoking before age 16 were less likely to quit than those who started later. Higher education and income levels were associated with higher odds of smoking cessation, while irregular employment and remote residence were associated with lower quitting rates. Married individuals had the highest cessation rates, whereas those who were unmarried or divorced were least likely to quit. Poorer self-rated health and the presence of long-term illness were associated with higher odds of smoking cessation, as well (*p* < 0.001).

**Table 1:** Bivariate comparisons of smoking cessation status across socio-economic and demographic characteristics.

**Variables**	**Category**	**Smoking status**		*P*-Value^*^
		**Active smoker** ***N*** **(%)**	**Smoking cessation** ***N*** **(%)**	
Gender	Male	756 (55.1)	617 (44.9)	**< 0.001**
	Female	722 (62.4)	436 (37.6)	
Age group	15–17 years old	30 (100)	0 (0)	**< 0.001**
	18–34 years old	410 (79.2)	108 (20.8)	
	35–64 years old	830 (62.3)	502 (37.7)	
	65 years and older	208 (31.9)	443 (68.1)	
Age start smoking	Younger than 16	352 (65.2)	188 (34.8)	**< 0.001**
	16 years and older	1,126 (56.6)	865 (43.4)	
Education	Primary	401 (75.2)	132 (24.8)	**< 0.001**
	Secondary	897 (57.2)	672 (42.8)	
	High	180 (42.0)	249 (58.0)	
Income level	Low	451 (70.8)	186 (29.2)	**< 0.001**
	Lower middle	315 (59.2)	217 (40.8)	
	Middle	250 (59.9)	167 (40.1)	
	Higher middle	214 (45.2)	259 (54.8)	
	High	248 (52.5)	224 (47.5)	
Employment status	Regular employment	836 (65.4)	442 (34.6)	**< 0.001**
	Irregular employment	165 (79.7)	42 (20.3)	
	Retired/disabled	324 (38.5)	518 (61.5)	
	Student/childcare	153 (75.0)	51 (25.0)	
Degree of urbanization	Urban	173 (48.7)	182 (51.3)	**< 0.001**
	Suburban	247 (52.5)	223 (47.5)	
	Rural	484 (56.1)	379 (43.9)	
	Remote	574 (68.1)	269 (31.9)	
Marital status	Registered marriage	583 (48.4)	622 (51.6)	**< 0.001**
	Unmarried partner	187 (69.3)	83 (30.7)	
	No marriage	394 (79.4)	102 (20.6)	
	Divorced	151 (59.7)	102 (40.3)	
	Widowed	120 (49.2)	124 (50.8)	
Health status	Good or excellent	882 (63.8)	501 (36.2)	**< 0.001**
	Satisfactory	438 (51.7)	409 (48.3)	
	Bad or very bad	150 (51.7)	140 (48.3)	
Long term illness	Yes	648 (50.7)	631 (49.3)	**< 0.001**
	No	809 (66.6)	405 (33.4)	

^*^Bold values indicate statistical significance (*p* < 0.05) based on Pearson's chi-squared test.

Table 2 presents the results of a logistic regression model assessing the odds of smoking cessation associated with socio-economic and demographic factors. Gender significantly associated with cessation, with females having lower odds of quitting compared to males (*OR* = 0.69, 95% CI: 0.56–0.84, *p* = 0.001), indicating that women were approximately 31% less likely to cease smoking. Age group was significantly associated with smoking cessation. Compared with the 16–17-year-old reference group, individuals aged 18–34 years had markedly lower odds of cessation (*OR* = 0.24, 95% CI: 0.16–0.38, *p* < 0.001), and those aged 35 years and older also showed lower odds of cessation (*OR* = 0.41, 95% CI: 0.29–0.57, *p* < 0.001). This suggests that younger individuals had lower odds of smoking cessation than the reference group, though cessation increased with age beyond adolescence. Compared to primary education, secondary education increased the odds of cessation nearly twofold (*OR* = 1.89, 95% CI: 1.45–2.87, *p* < 0.001), and higher education tripled the odds (*OR* = 3.07, 95% CI: 2.15–4.39, *p* < 0.001). The higher-middle income group had significantly higher odds of cessation compared to the low-income reference (*OR* = 1.95, 95% CI: 1.44–2.63, *p* = 0.001). Compared to regular employment, retired or disabled individuals had 57% higher odds of quitting (*OR* = 1.57, 95% CI: 1.16–2.14, *p* = 0.004). The degree of urbanization revealed that remote area residents had lower odds of cessation compared to urban areas (*OR* = 0.7, 95% CI: 0.52–0.98, *p* = 0.025). Compared to registered marriage, all other categories had reduced odds of cessation: unmarried partners (*OR* = 0.72, *p* = 0.049), no marriage (*OR* = 0.43, *p* < 0.001), divorced (*OR* = 0.58, *p* = 0.001), and widowed (*OR* = 0.62, *p* = 0.025).

**Table 2:** Logistic regression model.

**Variables**		**OR**	**95%CI**	***P*-Value**
Gender	Male (Reference)	–	–	
	Female	**0.69**	**0.56–0.84**	**0.001**
Age group	16–17 years old (Reference)	–	–	–
	18–34 years old	**0.24**	**0.16–0.38**	**< 0.001**
	Older than 35	0.41	0.29–0.57	< 0.001
Age start smoking	Younger than 16 (Reference)	–	–	–
	16 years and older	1.03	0.81–1.29	0.763
Education	Primary (reference)	–	–	–
	Secondary	**1.89**	**1.45–2.87**	**< 0.001**
	Higher	**3.07**	**2.15–4.39**	**< 0.001**
Income level	Low (reference)	–	–	–
	Lower middle	1.32	0.99–1.75	0.052
	Middle	1.04	0.76–1.41	0.789
	Higher middle	**1.95**	**1.44–2.63**	**0.001**
	High	1.38	0.99–1.91	0.053
Employment status	Regular employment (reference)	–	–	–
	Irregular employment	0.89	0.59–1.33	0.587
	Retired/disabled	**1.57**	**1.16–2.14**	**0.004**
	Student/childcare	1.48	0.98–2.24	0.058
Degree of urbanization	Urban (reference)	–	–	–
	Suburban	0.97	0.71–1.34	0.896
	Rural	1.06	0.8–1.42	0.656
	Remote	**0.7**	**0.52–0.98**	**0.025**
Marital status	Registered marriage (reference)	–	–	–
	Unmarried partner	**0.72**	**0.53–0.99**	**0.049**
	No marriage	**0.43**	**0.32–0.58**	**< 0.001**
	Divorced	**0.58**	**0.42–0.79**	**0.001**
	Widowed	**0.62**	**0.52–0.95**	**0.025**
Health status Long-term illness	Good or excellent (reference)	–	–	–
	Satisfactory	0.94	0.74–1.19	0.633
	Bad or very bad	1.03	0.73–1.46	0.846
	Yes (reference)	–	–	–
	No	0.84	0.67–1.05	0.129

Bold values indicate statistical significance (*p* < 0.05). Odds ratios (OR) are adjusted for variables in the model. AUC = 0.707 (95% CI 0.668–0.746).

Table 3 presents the results of weighted logistic regression models examining the interactions between socio-demographic factors, including age, sex, education, and income, in predicting smoking cessation. In Model 1, adolescent females aged 15–17 years had more than twice the odds of smoking cessation compared to males of the same age (*OR* = 2.34, 95% CI: 1.30–4.20, *p* = 0.004), while women aged 35–64 years also had higher odds of quitting than men in the same age group (*OR* = 1.91, 95% CI: 1.24–2.95, *p* = 0.003). Model 2 indicated a significant interaction between education level and age group in relation to smoking cessation. Among individuals aged 18–34 years, those with secondary education (*OR* = 3.36, 95% CI: 1.39–8.12, *p* = 0.007) or higher education (*OR* = 3.24, 95% CI: 1.15–9.17, *p* = 0.026) had higher odds of smoking cessation than those with only primary education. Similarly, adults aged 35–64 with secondary education (*OR* = 1.80, 95% CI: 1.03–3.15, *p* = 0.038) or higher education (*OR* = 2.31, 95% CI: 1.14–4.66, *p* = 0.019) showed increased odds of cessation. Model 3 indicated that income level was significantly associated with smoking cessation across age groups. Among participants aged 18–34 years, those with middle income (*OR* = 2.86, 95% CI: 1.13–7.23, *p* = 0.026), middle-high income (*OR* = 3.54, 95% CI: 1.43–8.77, *p* = 0.006), or high income (*OR* = 3.42, 95% CI: 1.21–9.63, *p* = 0.020) had higher odds of smoking cessation compared with those in the low-income group. Among adults aged 35–64 years, middle-high income was also associated with higher odds of smoking cessation (*OR* = 3.32, 95% CI: 1.75–6.28, *p* < 0.001). Overall, these findings indicate that higher education and income levels were associated with higher odds of smoking cessation, particularly among younger adults. In addition, females in certain age groups showed higher odds of cessation than males, highlighting socio-demographic differences in cessation patterns.

**Table 3:** Logistic regression models predicting smoking cessation with interaction terms.

**Interaction Term**	**Model**	**OR**	**95% CI**	***p* – Value**
15–17 years old female/male	Model 1	2.34	1.30–4.20	**0.004**
35–64 years old female/male	Model 1	1.91	1.24–2.95	**0.003**
18–34 years old, secondary education/primary education	Model 2	3.36	1.39–8.12	**0.007**
35–64 years old, secondary education/primary education	Model 2	1.80	1.03–3.15	**0.038**
18–34 years old, higher education/primary education	Model 2	3.24	1.15–9.17	**0.026**
35–64 years old, higher education/primary education	Model 2	2.31	1.14–4.66	**0.019**
18–34 years old, middle income/low income	Model 3	2.86	1.13–7.23	**0.026**
18–34 years old, middle-high income/low income	Model 3	3.54	1.43–8.77	**0.006**
18–34 years old, high income/low income	Model 3	3.42	1.21–9.63	**0.02**
35–64 years old, middle-high income/low income	Model 3	3.32	1.75–6.28	**< 0.001**

OR = Odds ratio; 95% CI = 95% confidence interval. Model 1: Interaction between age group and gender; Model 2: Interaction between age group and education; Model 3: Interaction between age group and income levels. All models are adjusted for the same variables in the weighted logistic regression model shown in Table 2.

## 4 Discussions

The objective of this study was to examine socio-demographic and economic factors associated with smoking cessation among Hungarian adults. The results identify key demographic and socio-economic variables associated with smoking cessation Hungary, providing a basis for understanding disparities in quitting success.

The results indicate that females are significantly less likely to quit smoking compared to males (*OR* = 0.69, *p* = 0.001), indicating gender-specific differences in smoking cessation patterns. Research indicates that gender plays a significant role in smoking cessation outcomes. Women generally achieve lower cessation rates than men, both with and without pharmacotherapy (19). Psychological and behavioral factors also impact cessation, with women being more vulnerable to depression, negative mood, and weight concerns (20). The consistency of gender differences in cessation rates in our study highlights the need for further research to elucidate the underlying mechanisms and develop gender-specific interventions.

Literature on age-related factors in smoking cessation reveals complex patterns. Older adults show higher success rates when using nicotine replacement therapy alone compared to younger smokers (21). Age-specific factors have been associated with differences in cessation outcomes, with marriage positively associated in young and middle-aged groups, while willpower and comorbidities are more relevant for middle-aged and older adults (22). The incidence of long-term cessation follows a cubic trend, with higher rates in young (21–30) and older (51–60) adults compared to middle-aged (31–50) individuals (23). Age was significantly associated with smoking cessation according to our results, with both younger and middle-aged groups showing lower odds of quitting compared to the 16–17-year-olds. The observed differences in cessation across age groups underscore the potential public health benefits of encouraging earlier cessation, although causal inferences cannot be drawn from this cross-sectional analysis.

Socio-economic status, particularly education, was strongly associated with smoking cessation. Research consistently shows that higher education levels are associated with lower smoking rates and increased likelihood of smoking cessation. College-educated individuals among young adults are less likely to smoke and more likely to quit if they do smoke (24). This educational gradient in smoking behavior persists even after controlling for demographic and health-related variables (25). The impact of education on smoking cessation becomes particularly pronounced following significant health events like heart attacks, with more educated individuals being dramatically more likely to quit smoking after such incidents (26). The results of our study show that individuals with secondary and higher education were significantly more likely to quit compared to those with only primary education. This finding is consistent with the notion that higher education may be linked to differences in health literacy and risk awareness, which could be related to smoking cessation patterns. It highlights the complex relationship between education and smoking behaviors, emphasizing the need for targeted interventions across educational groups.

Income levels were significantly associated with smoking cessation outcomes. Low-income smokers have lower odds of achieving 6-month continuous abstinence compared to middle- or high-income smokers (27). This can be explained by both material and psychosocial mechanisms contributing to observed disparities. The materialist theory suggests that individuals with higher incomes possess better access to tangible resources such as quality healthcare, cessation aids, and supportive environments, all of which are critical for quitting attempts (28). The psychosocial theory further explains how income levels indirectly influence smoking cessation through factors such as social status, stress, and access to social support networks. Financial strain can heighten stress and lower self-efficacy for quitting, thus weakening cessation efforts (29). Our findings are consistent with these theoretical frameworks by demonstrating a strong link between income levels and successful smoking cessation, suggesting that financial resources may enhance access to cessation aids (e.g., nicotine replacement therapy) or mitigate stress-related smoking triggers.

Employment status significantly influences smoking cessation outcomes, with complex interactions observed across gender and socioeconomic factors. Unemployed individuals generally have lower quit rates compared to employed smokers (30). However, gender differences exist, with employed men showing higher quit rates than unemployed men, while employed women have lower quit rates than unemployed women (31). Interestingly, part-time workers and multiple job holders have higher odds of being former smokers compared to full-time workers and single job holders (32). Our study results did not show a significant difference between employed and unemployed people, but retired or disabled individuals had higher odds of smoking cessation compared to those with regular employment. This could reflect increased motivation due to health concerns or more time to engage with cessation programs.

In addition, urbanization was significantly associated with smoking cessation patterns, with higher cessation rates observed in more urbanized areas (33). However, disparities exist within urban settings, as younger urban populations are less likely to receive smoking cessation counseling compared to older individuals (34). Environmental smoking restrictions have been associated with higher smoking cessation rates in previous studies, with city-level public places and workplace restrictions, as well as individual-level workplace and household restrictions, positively associated with quitting success (35). Our study also found a similar pattern, as population density decreases, the likelihood of quitting smoking decreases. This can be explained in the same way as above, indicating that a legal framework for tobacco control that prohibits smoking in public places is important in increasing the likelihood of quitting smoking.

Research indicates that marital status significantly influences smoking cessation. Married individuals, particularly women, are more likely to quit smoking compared to unmarried or divorced individuals (36). The smoking habits of a partner play a crucial role, with non-smoking partners or those who quit smoking increasing the likelihood of cessation (37). Our research results showed that people living alone or without a legally recognized partner, such as those who are unmarried, divorced, or widowed, are less likely to quit smoking than those with a registered partner.

As previously discussed, smoking is a well-established risk factor for numerous diseases. Therefore, the presence of chronic health conditions may serve as a significant motivator for smoking cessation. Some studies found that certain chronic diseases, particularly diabetes, were associated with a higher likelihood of being a former smoker (38). Interestingly, neither health status nor the presence of a long-term illness significantly influenced quitting likelihood, suggesting that perceived health or chronic diseases alone may not be a strong motivator in this population. Overall, while chronic disease diagnoses have been associated with smoking cessation, their effects may be limited, suggesting the need for targeted interventions.

While this study provides important insights, its implications should be considered in light of several methodological limitations. The cross-sectional design of the 2019 EHIS dataset prevents any causal inference regarding the relationship between socio-economic characteristics and smoking cessation; for example, although higher education is associated with greater odds of quitting, the directionality of this relationship cannot be confirmed. Smoking status was self-reported and defined as point prevalence abstinence at the time of the interview, which may overestimate long-term cessation. Additionally, abstinence was assessed for cigarette smoking only, as information on other nicotine-containing products was insufficient for detailed classification. The reliance on self-reported measures may also introduce recall or social desirability bias, particularly in socio-economic reporting. Additionally, certain potentially relevant factors, such as detailed smoking history, psychosocial influences, or exposure to cessation support, were not captured in the dataset.

Despite these limitations, the study has several notable strengths. It draws on a large, nationally representative sample, supporting strong statistical power and enhancing generalizability to the Hungarian adult population. The inclusion of a broad set of socio-economic indicators, including education, income, employment, urbanization, and marital status, allows for a comprehensive assessment of social determinants that have been underexplored in previous Hungarian research. Finally, the use of weighted multivariable logistic regression, including interaction terms and adjustment for key confounders, ensures a rigorous analytical approach and strengthens the robustness of the findings.

## 5 Conclusion

These findings indicate that smoking cessation is strongly associated with socio-demographic factors. Females, individuals with higher education, and those with greater income levels were associated with significantly higher odds of smoking cessation, particularly among younger and middle-aged adults. These findings highlight the need for targeted interventions, such as gender-tailored support for women, age-targeted interventions, and expanded cessation resources in remote areas, as well as efforts to reduce educational and financial inequalities to address Hungary's high smoking prevalence and align with global tobacco control objectives. Future longitudinal studies should examine how access to cessation programs, mental health, and broader social or environmental factors shape quitting behavior.

## Data Availability

The data analyzed in this study is subject to the following licenses/restrictions. The data presented in this study are available upon request from Hungarian Central Statistical Office who performed and supervised the data collection. Requests to access these datasets should be directed to Hungarian Central Statistical Office. Requests to access these datasets should be directed to https://www.ksh.hu/?lang=en.
